# Flotillin 2 Facilitates the Infection of a Plant Virus in the Gut of Insect Vector

**DOI:** 10.1128/jvi.02140-21

**Published:** 2022-03-07

**Authors:** Wei Wang, Luqin Qiao, Hong Lu, Xiaofang Chen, Xue Wang, Jinting Yu, Jiaming Zhu, Yan Xiao, Yonghuan Ma, Yao Wu, Wan Zhao, Feng Cui

**Affiliations:** a State Key Laboratory of Integrated Management of Pest Insects and Rodents, Institute of Zoology, Chinese Academy of Sciences, Beijing, China; b College of Plant Protection, Shandong Agricultural University, Tai’an, Shandong, China; c CAS Center for Excellence in Biotic Interactions, University of Chinese Academy of Sciences, Beijing, China; d State Key Laboratory of Plant Genomics, Institute of Microbiology, Chinese Academy of Sciences, Beijing, China; Emory University School of Medicine

**Keywords:** flotillin 2, insect vector, midgut, planthopper, plasma membrane protein, rice stripe virus, rice viral disease

## Abstract

Most plant viruses require insect vectors for transmission. One of the key steps for the transmission of persistent-circulative plant viruses is overcoming the gut barrier to enter epithelial cells. To date, little has been known about viral cofactors in gut epithelial cells of insect vectors. Here, we identified flotillin 2 as a plasma membrane protein that facilitates the infection of rice stripe virus (RSV) in its vector, the small brown planthopper. Flotillin 2 displayed a prominent plasma membrane location in midgut epithelial cells. The nucleocapsid protein of RSV and flotillin 2 colocalized on gut microvilli, and a nanomolar affinity existed between the two proteins. Knockout of *flotillin 2* impeded the entry of virions into epithelial cells, resulting in a 57% reduction of RSV levels in planthoppers. The knockout of *flotillin 2* decreased disease incidence in rice plants fed by viruliferous planthoppers from 40% to 11.7%. Furthermore, flotillin 2 mediated the infection of southern rice black-streaked dwarf virus in its vector, the white-backed planthopper. This work implies the potential of flotillin 2 as a target for controlling the transmission of rice stripe disease.

**IMPORTANCE** Plant viral diseases are a major threat to world agriculture. The transmission of 80% of plant viruses requires vector insects, and 54% of vector-borne plant viruses are persistent-circulative viruses, which must overcome the barriers of gut cells with the help of proteins on the cell surface. Here, we identified flotillin 2 as a membrane protein that mediates the cell entry of rice stripe virus in its vector insect, small brown planthopper. Flotillin 2 displays a prominent cellular membrane location in midgut cells and can specifically bind to virions. The loss of flotillin 2 impedes the entry of virions into the midgut cells of vector insects and substantially suppresses viral transmission to rice. Therefore, *flotillin 2* may be a promising target gene for manipulation in vector insects to control the transmission of rice stripe disease and perhaps that of other rice virus diseases in the future.

## INTRODUCTION

Plant viral diseases are a major threat to world agriculture. The transmission of 80% of plant viruses requires vector insects (1), and 54% of vector-borne plant viruses are of the persistent-circulative type (2). These viruses exhibit the most complex transmission process inside vector insects, entering into gut epithelial cells from the gut lumen, then being released into the hemolymph and spreading to other organs, including the salivary glands; and finally, being secreted from the salivary glands into host plants (3). Considerable barriers to virus transmission exist in the first step of virus entry into gut cells. The ability of viruses to interact with surface factors of gut cells has long been recognized as a major determinant of vector competence for a variety of viruses. Unfortunately, little is known about the specific proteins on the plasma membrane of gut cells in vector insects which facilitate the transmission of persistent circulative plant viruses.

Rice stripe virus (RSV) is a nonenveloped negative-strand RNA virus of the *Tenuivirus* genus (4). This virus causes the most destructive rice stripe disease, inducing up to 80% disease incidence and 30 to 40% yield losses in the rice fields of Asian countries (5, 6). The genome of RSV consists of four RNA segments, encoding one nucleocapsid protein (NP), one RNA-dependent RNA polymerase (RdRp), and five nonstructural proteins (7). NP localizes to the exterior of the viral particle and encapsidates each viral genomic RNA molecule to form 8-nm-wide filaments, which frequently fold to form pleomorphic or branched configurations (4, 8). RSV is efficiently transmitted by the small brown planthopper, Laodelphax striatellus, in a persistent, circulative, and propagative way. Like most insect-transmitted persistent plant viruses, RSV overcomes the midgut barrier during the first step and establishes complicate interactions with planthoppers during the circulative process (9–12). In addition to sugar transporter 6, which has been proved to be helpful for RSV entry into the midgut cells of planthoppers (13), no other midgut cell membrane proteins have been identified as possible receptors of RSV. In our recent work, we predicted protein-protein interactions between five rice viruses [RSV, southern rice black-streaked dwarf virus (SRBSDV), rice ragged stunt virus (RRSV), rice black-streaked dwarf virus (RBSDV), and rice grassy stunt virus (RGSV)] and their respective vector insects (*L. striatellus*, *Nilaparvata lugens*, and *Sogatella furcifera*) on a genome-wide scale using the *de novo* method and provided a list of potential conserved vector proteins that are essential for the transmission of rice viruses (14). Flotillin 2 is one of these conserved vector proteins, and the interaction between the RSV NP and flotillin 2 of small brown planthopper was verified *in vitro* (14).

Flotillins are membrane proteins that form microdomains in the plasma membranes of mammalian cells. The flotillin family is composed of two highly homologous proteins, flotillin 1 and flotillin 2, characterized by the presence of a prohibitin homology (PHB) domain, which is responsible for membrane association (15, 16). Flotillin-dependent endocytosis has been suggested to mediate the internalization of nonvirus cargo molecules, such as the GPI-anchored protein CD59, the cholera toxin B subunit, and proteoglycans (17). Whether viruses can exploit flotillins for cell entry has not yet been reported.

In this study, we explored the functions of flotillins in the entry of RSV into the midgut epithelial cells of vector insects. One flotillin, flotillin 2, was found to play a key role in enabling RSV to overcome the midgut barrier and affected the vector competence of the small brown planthopper for RSV.

## RESULTS

### Characterization of small brown planthopper *flotillins*.

**FIG 1:**
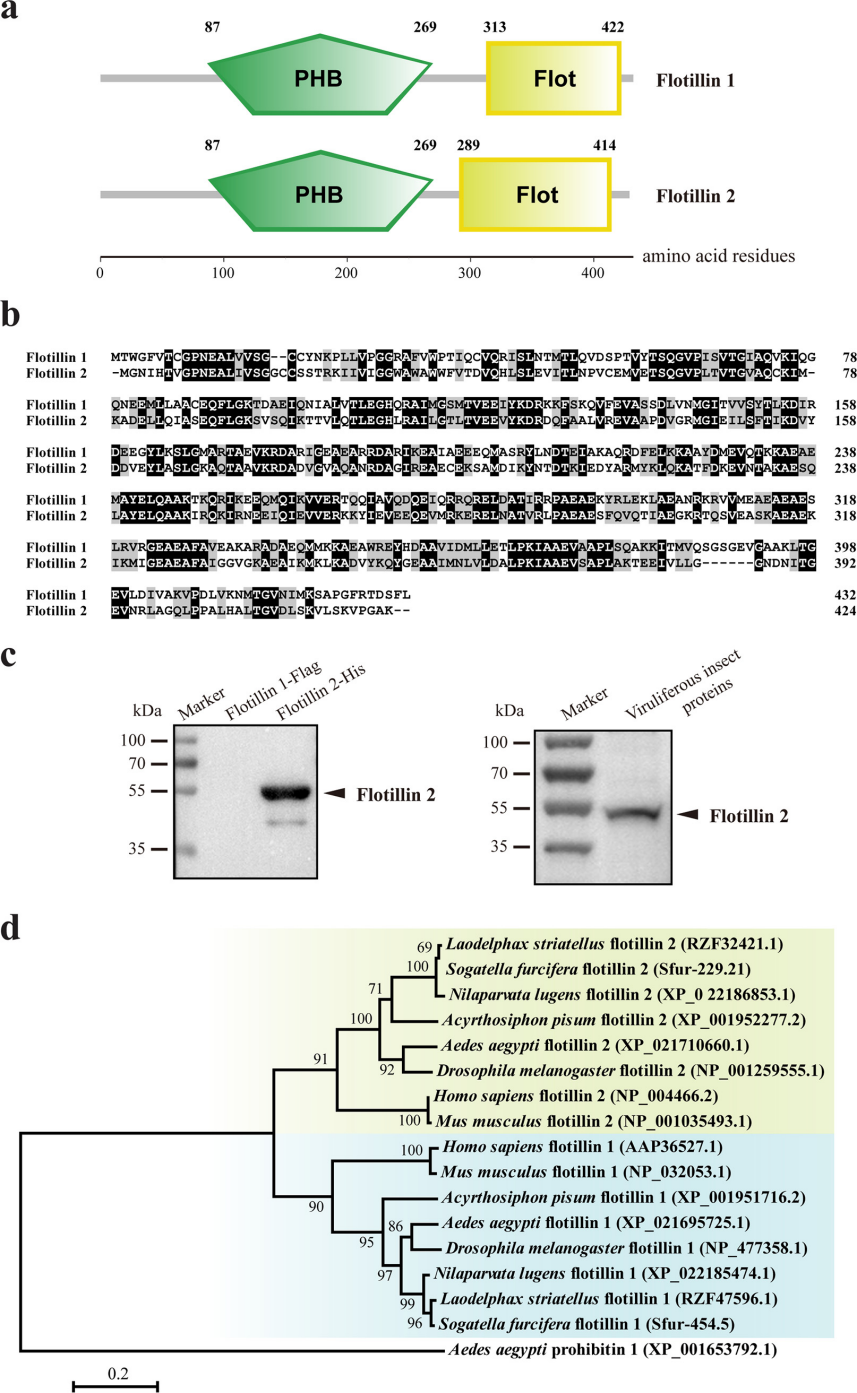
Characterization of small brown planthopper *flotillins*. (a) Diagram showing the PHB and flotillin (Flot) domains of flotillin 1 and flotillin 2. (b) Alignment of amino acid sequences of flotillin 1 and flotillin 2. Similar amino acid residues are shaded in gray. Identical amino acid residues are shaded in black. (c) Verification of anti-flotillin 2 polyclonal antibody in the recombinantly expressed flotillin 2-His and flotillin 1-Flag and the total protein of viruliferous planthoppers in Western blot assays. (d) Phylogenetic neighbor-joining tree of flotillins of the small brown planthopper and other insects and mammals. Bootstrap values higher than 60% are shown at the nodes. GenBank registration numbers of each protein are given in parentheses.

Two *flotillin* genes, *flotillin 1* (evm.TU.Contig589.22) and *flotillin 2* (evm.TU.Contig20.196), were identified from the gene set of the small brown planthopper (18). The open reading frame (ORF) of *flotillin 1* was 1,299 bp, and putatively encoded a 48.1-kDa protein with 432 amino acid (aa) residues. The typical membrane association domain PHB was predicted to extend from 87 to 269 aa, and the flotillin domain was predicted to extend from 313 to 422 aa (Fig. 1a). The ORF of *flotillin 2* was 1,275 bp, encoding a 46.3-kDa protein with 424 aa. Flotillin 2 also contained the conserved PHB (from 87 to 269 aa) and flotillin (from 289 to 414 aa) domains (Fig. 1a). The two flotillins showed 45% identity in their amino acid sequences (Fig. 1b). The anti-flotillin 2 polyclonal antibody we made showed the specific recognition of flotillin 2 and not of flotillin 1 (Fig. 1c). The two flotillins of the small brown planthopper were most closely related to their respective homologs from the brown planthopper, *N. lugens*, and the white-backed planthopper, *S. furcifera*, and they formed two orthologous clusters with the homologs from other insects and mammals in the phylogenetic analysis (Fig. 1d).

### Flotillin 2 binds to RSV NP with specific affinity activity.

**FIG 2:**
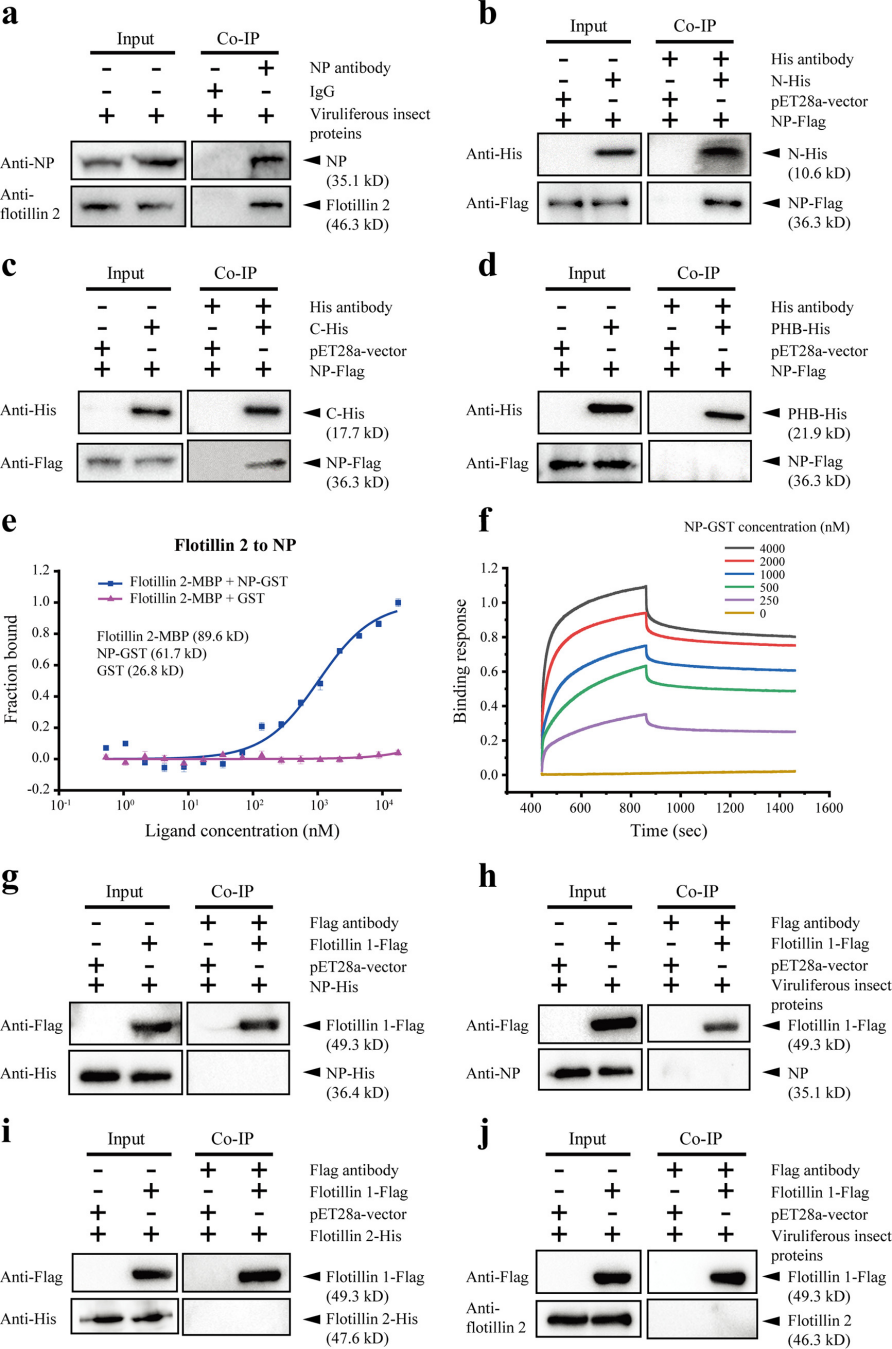
Flotillin 2 binds to RSV NP with specific affinity activity. (a) NP and flotillin 2 were coprecipitated from the total protein of viruliferous planthoppers using anti-NP monoclonal antibody and analyzed in Western blot. Mouse IgG was used as a negative control. (b) The recombinantly expressed N terminus of flotillin 2 with His tags (N-His) was coprecipitated with NP-Flag. The total protein from E. coli expressing the empty pET28a vector was applied as a negative control. (c) The C terminus of flotillin 2 with His-tags (C-His) was coprecipitated with NP-Flag. (d) The PHB domain of flotillin 2 with His-tags (PHB-His) was not coprecipitated with NP-Flag. (e) MST assay to reveal specific binding between NP-GST and flotillin 2-MBP. Solid curve shows fit of data points to a standard *K*_D_-fit function. Bars represent standard error (SE). (f) BLI binding profiles of NP-GST and flotillin 2-MBP. (g and h) Flotillin 1-Flag was not coprecipitated with NP-His (g) or NP from total protein of the viruliferous planthoppers (h). (i and j) Flotillin 1-Flag was not coprecipitated with flotillin 2-His (i) or flotillin 2 from the total protein of the viruliferous planthoppers (j).

Our recent study provided basic clues regarding the interaction between RSV NP and flotillin 2 using *in vitro*-expressed recombinant proteins (14). Here, we obtained NP and flotillin 2 from viruliferous planthoppers using an anti-NP monoclonal antibody in coimmunoprecipitation (Co-IP) assays (Fig. 2a), confirming the interaction of these two proteins *in vivo*. To explore the specific region of flotillin 2 which interacted with NP, we recombinantly expressed the N termini, PHB domains, and C termini of flotillin 2 and NP. The *in vitro* Co-IP assays showed that the N and C termini of flotillin 2 were able to bind to NP (Fig. 2b and c), while the PHB membrane association domain was not (Fig. 2d). Specific affinity activity between flotillin 2 and NP was measured using both microscale thermophoresis (MST) and bio-layer interferometry (BLI) assays with the MBP-tagged flotillin 2 and GST-tagged NP. The dissociation constant (*K*_D_) from the MST assay was 791 ± 235 nM (Fig. 2e). A similar binding affinity (*K*_D_ = 619 ± 233 nM) was obtained from the BLI assay, which also estimated the binding kinetics parameters, the association rate *K*_on_ = 6169 ± 375 M^−1^s^−1^ and the dissociation rate *K*_off_ = 3.82 ± 1.42 × 10^−3^ s^−1^ (Fig. 2f). These data show that flotillin 2 specifically binds to RSV NP with a nanomolar affinity.

We also tested the interaction between flotillin 1 and NP or flotillin 2. The recombinantly expressed flotillin 1-Flag did not bind to NP-His or NP from viruliferous planthoppers, and it did not bind to flotillin 2-His or flotillin 2 from viruliferous planthoppers in Co-IP assays (Fig. 2g to j).

### Organ and plasma membrane localization of *flotillin 2*.

**FIG 3:**
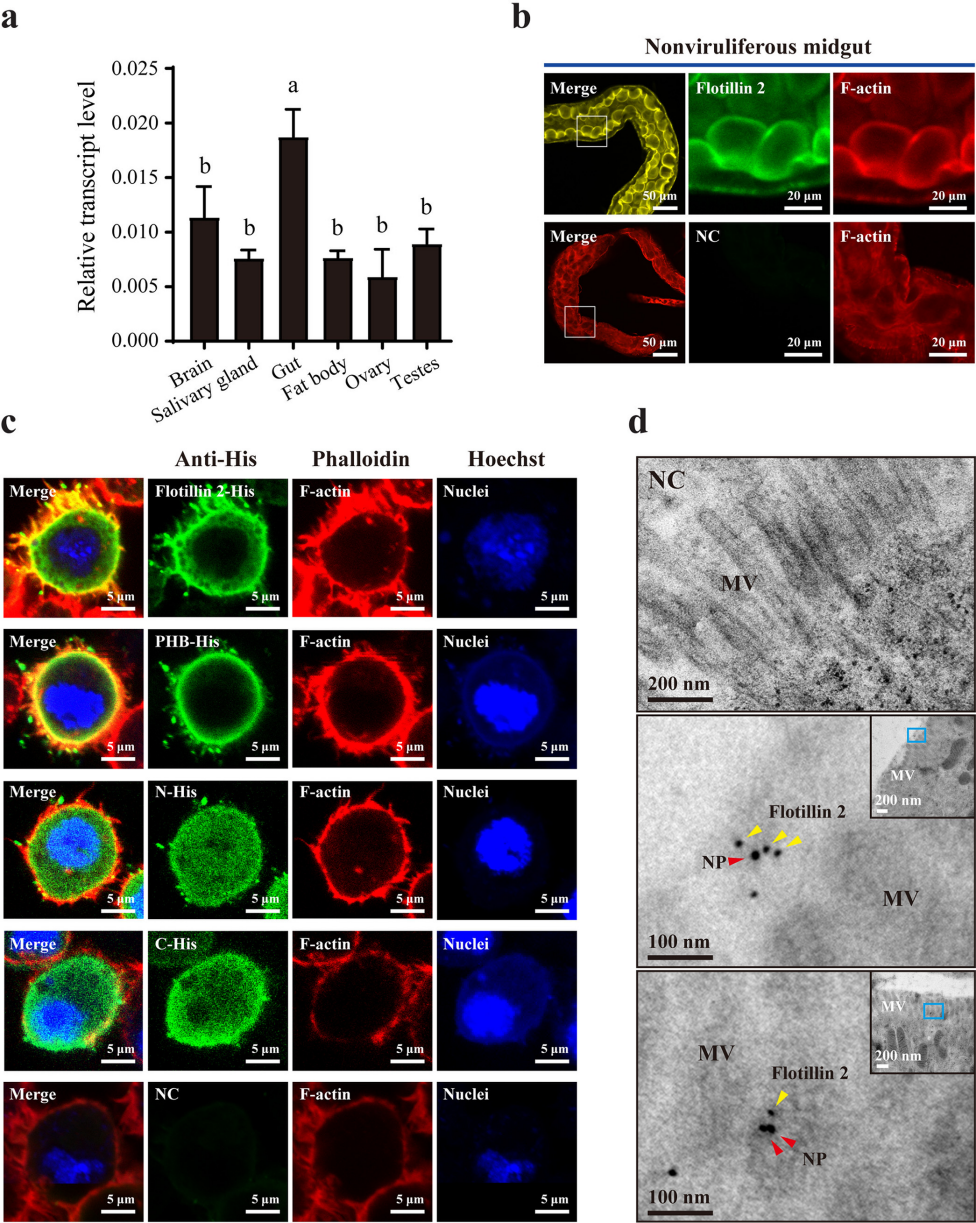
Organ and plasma membrane localization of *flotillin 2*. (a) Relative transcript levels of *flotillin 2* in various organs of nonviruliferous small brown planthopper after normalization according to the transcript level of EF2. Different letters indicate statistically significant differences. (b) Immunohistochemistry showing localization of flotillin 2 in planthopper midgut epithelial cells. Boxed regions are enlarged and shown in the two panels on the right. Flotillin 2 was labeled with anti-flotillin 2 polyclonal antibody (red). F-actin was stained with phalloidin (blue). The sample without antibody treatment is shown as negative control (NC). (c) Immunofluorescence labeling of recombinantly expressed flotillin 2-His, PHB-His domain, N-terminal-His (N-His), and C-terminal-His (C-His) of flotillin 2 in *Drosophila* S2 cells with anti-His monoclonal antibody (green). F-actin was labeled with phalloidin (red) and nuclei with Hoechst (blue). (d) Colloidal gold immunoelectron micrographs showing the colocalization of flotillin 2 and RSV NP on the microvilli (MV) of midgut epithelial cells. Images include enlarged images of the regions in blue boxes in the upper right corner panels. NP was labeled with the 10-nm colloidal gold-conjugated anti-NP monoclonal antibody, and flotillin 2 with the 5-nm colloidal gold-conjugated anti-flotillin 2 polyclonal antibody.

Considering that only flotillin 2 bound RSV NP, we explored the expression profile of *flotillin 2* in various organs and the subcellular localization of its protein in nonviruliferous planthoppers. Real-time quantitative PCR (RT-qPCR) demonstrated that *flotillin 2* exhibited broad expression in various organs, showing higher expression in the gut than in the brain, salivary gland, ovary, testicle, and fat body (Fig. 3a). Flotillin 2 was mainly localized on the plasma membrane of midgut epithelial cells (Fig. 3b). To further confirm the subcellular localization of the protein, the full length, N terminus (1 to 86 aa), PHB domain (87 to 269 aa), and C terminus (270 to 424 aa) of flotillin 2 were recombinantly expressed with His tags in *Drosophila* S2 cells. Immunofluorescence demonstrated that flotillin 2 was distributed mainly on the plasma membrane and that the PHB domain showed a similar distribution pattern to that of the intact protein (Fig. 3c). In contrast, the N and C termini of flotillin 2 localized in the cytoplasm and did not present a typical membrane distribution (Fig. 3c). Therefore, the PHB domain is responsible for the membrane localization of flotillin 2.

In the midgut epithelial cells of viruliferous planthoppers, when NP and flotillin 2 were labeled with 10- and 5-nm colloidal gold particles, respectively, the two particle sizes were found to colocalize 87 times on the microvilli of 6 midguts in immunoelectron microscopy images (Fig. 3d), indicating that flotillin 2 bound RSV virions on the microvilli of midgut epithelial cells.

### Flotillin 2 facilitates RSV infection in small brown planthoppers.

**FIG 4:**
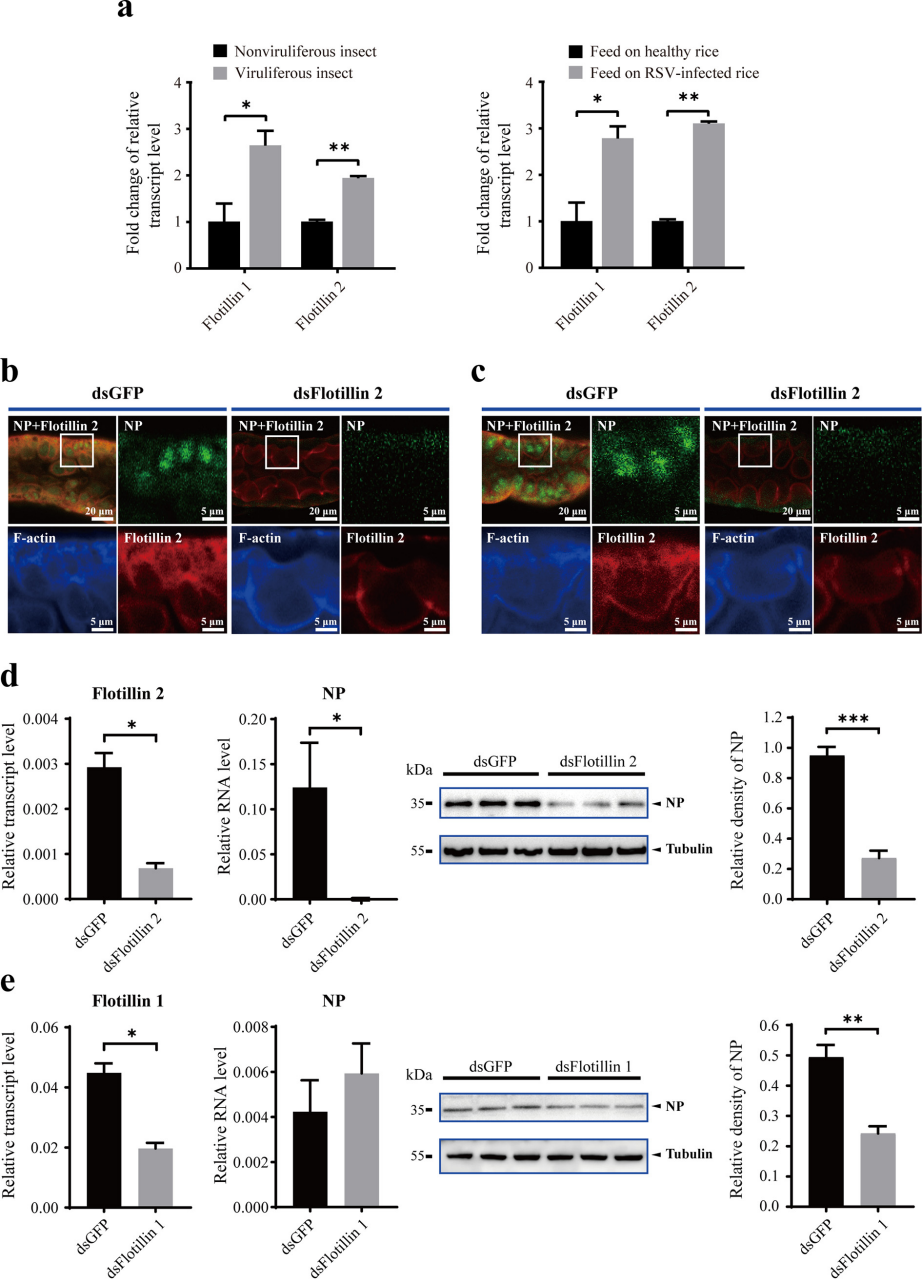
Flotillin 2 facilitates RSV infection in small brown planthoppers. (a) Comparison of relative *flotillin* gene transcription levels in the fourth-instar nymphs of viruliferous and nonviruliferous planthoppers, and in planthoppers fed on RSV-infected rice or uninfected rice for 8 days, as measured by qPCR. The flotillin gene transcription levels were normalized to that of EF2. Fold changes relative to those of nonvirulifous insects are shown. (b and c) Immunohistochemistry results showing the variation of RSV load in midgut epithelial cells after 12 h (b) or 10 days (c) of oral acquisition of RSV with the injection of dsRNA for *flotillin 2* (dsFlotillin 2) compared to the injection of *GFP* dsRNA (dsGFP). Boxed regions are enlarged and shown in other three panels. NP was labeled with anti-NP monoclonal antibody (green), flotillin 2 with anti-flotillin 2 polyclonal antibody (red), and F-actin with phalloidin (blue). Fifteen midguts were analyzed for each group. NP fluorescence intensity decreased by 77% (*P* < 0.01) (b) and 86.2% (*P* < 0.001) (c). (d and e) Relative RNA and protein levels of RSV *NP* in planthoppers after 10 days of oral acquisition of RSV with the injection of dsFlotillin 2 (d) or dsFlotillin 1 (e) as measured by qPCR and Western blot. Control groups were injected with dsGFP. Relative transcription levels of *flotillin 2* and *flotillin 1* were also measured. Anti-NP monoclonal antibody and anti-human β-tubulin monoclonal antibody were used. The relative grayscale of NP to that of tubulin was compared between groups. *, *P* < 0.05; **, *P* < 0.01; ***, *P* < 0.001.

The response and effect of the two *flotillin* genes toward RSV infection were checked. The transcription levels of both *flotillin 2* and *flotillin 1* were significantly upregulated in the whole bodies of viruliferous insects compared to nonviruliferous insects (Fig. 4a). The upregulation was also observed in the planthoppers at 8 days postinoculation (dpi) of RSV from infected rice plants (Fig. 4a).

When the expression of *flotillin 2* was knocked down with the injection of double-stranded RNA (dsRNA) targeting *flotillin 2*, fewer virions entered in the cytoplasm of midgut epithelial cells after 12 h of oral acquisition of RSV (Fig. 4b) and much lower viral load was observed in the midgut epithelial cells at 10 dpi (Fig. 4c) compared to the control group which was injected with green flourescent protein (GFP) dsRNA (dsGFP). The amount of RSV, in terms of RNA and NP levels, was much lower in the whole body at 10 dpi (Fig. 4d). On the other hand, the knockdown of *flotillin 1* using an injection of dsRNA targeting *flotillin 1* did not significantly affect viral levels in the whole body (Fig. 4e).

### Flotillin 2 is critical for the transmission of RSV by small brown planthoppers.

**FIG 5:**
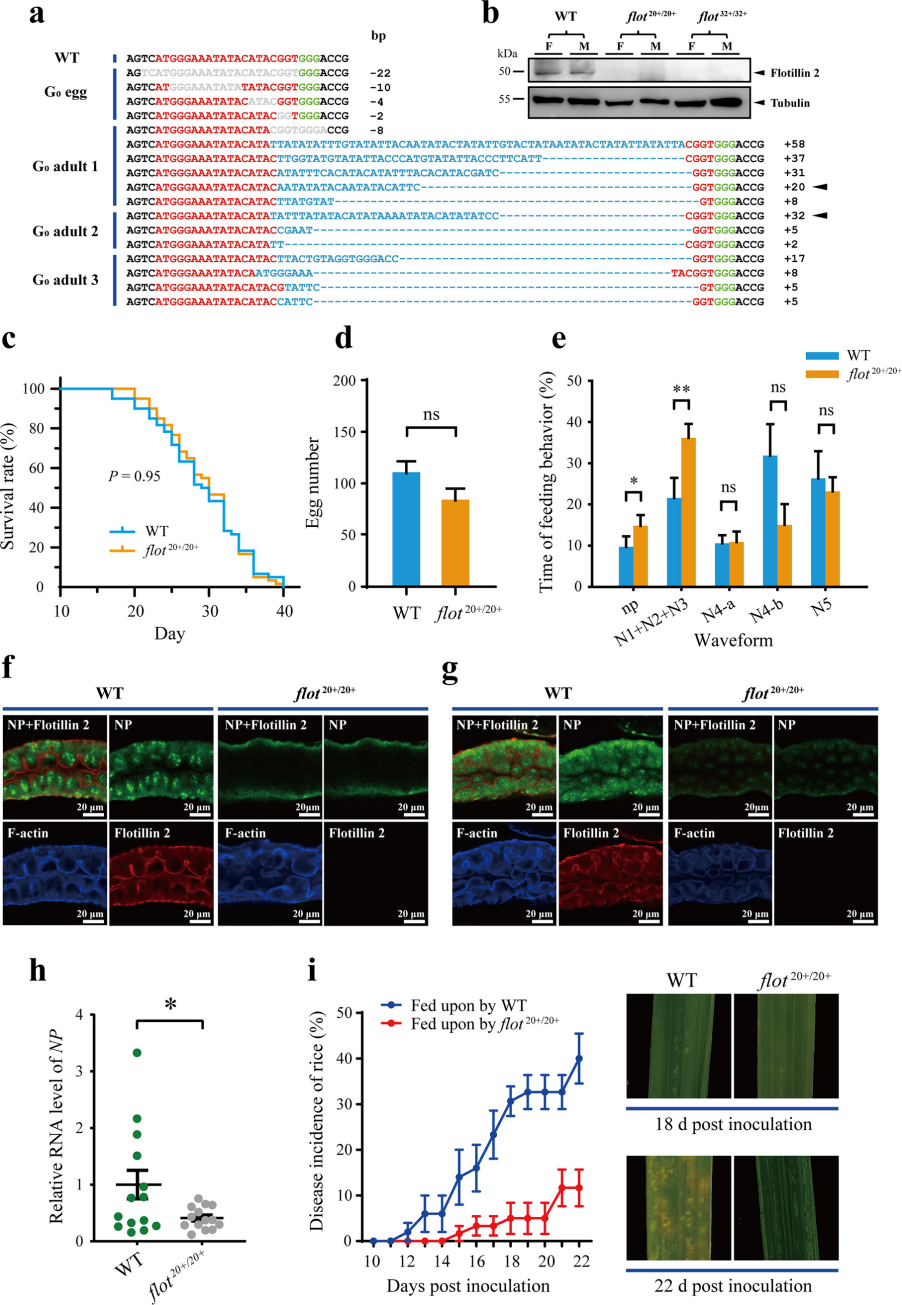
Flotillin 2 is critical for the transmission of RSV by small brown planthopper. (a) Germ-line insertion/deletion mutations in the *flotillin 2* gene in G_0_ eggs and adults generated using CRISPR-Cas9. Target sequence is indicated in red. PAM sequence is indicated in green. Deleted nucleotides are indicated in gray, and inserted nucleotides are in blue. The length of the nucleotide alteration is indicated on the right (+, insertion; –, deletion). Black triangles indicate the genotypes of two successfully established *flotillin 2*-knockout lines (*flot*^20+/20+^, *flot*^32+/32+^). WT, wild type. (b) Protein levels of flotillin 2 in the two homozygous mutant lines and in WT planthoppers, assayed by Western blot using the anti-flotillin 2 polyclonal antibody. F, female. M, male. (c) Survival curves of *flot*^20+/20+^ and WT planthoppers throughout whole life spans. (d) Average numbers of eggs produced by single females of *flot*^20+/20+^ or WT planthoppers within 10 days. (e) Comparison of feeding behavior EPG waveforms. np, non-probing; N1+N2+N3, pathway phases; N4-a, watery salivation; N4-b, passive ingestion; N5, drinking from xylem. (f and g) Immunohistochemistry results showing RSV load and *flotillin 2* expression in the midgut epithelial cells of *flot*^20+/20+^ and WT planthoppers after 12 h (f) or 15 days (g) of oral acquisition of RSV. NP was labeled with anti-NP monoclonal antibody (green), flotillin 2 with anti-flotillin 2 polyclonal antibody (red), and F-actin with phalloidin (blue). Eight midguts were analyzed for each group. The NP fluorescence intensity decreased by 79.4% (*P* < 0.01) (f) and 81.2% (*P* < 0.01) (g). (h) Relative RNA levels of RSV *NP* in single individuals of *flot*^20+/20+^ or WT planthoppers after 15 days of oral acquisition of RSV measured using qPCR. (i) The disease incidence and symptoms of the rice plants fed upon by *flot*^20+/20+^ or WT viruliferous planthoppers. *, *P* < 0.05; **, *P* < 0.01; ns, *P* > 0.05.

To systemically evaluate the roles of flotillin 2 in the transmission of RSV, we generated germ line mutations in the *flotillin 2* gene in the small brown planthopper using the CRISPR-Cas9 system. A single guide RNA (sgRNA) targeting the first 20 bp of the *flotillin 2* ORF and the Cas9 protein were microinjected into preblastoderm eggs. In the first batch of experiments, 150 eggs were injected, and 16 survived after 2 days. Sanger sequencing showed that 12 out of the 16 eggs harbored deletions of various lengths within the predicted cleavage site of *flotillin 2* (Fig. 5a), demonstrating the successful application of the CRISPR-Cas9 technique in the small brown planthopper. In the second batch of experiments, 730 eggs were injected, and after 10 days, 27 (4%) successfully hatched to first-instar nymphs, of which only 9 individuals survived to the adult stage (G_0_), including 5 females and 4 males. After mating with wild-type planthoppers to obtain G_1_ eggs, the G_0_ adults were individually analyzed for *flotillin 2* via Sanger sequencing. Three of the 9 adults exhibited a range of indels occurring in the predicted cleavage site, and the majority harbored insertions of various lengths (Fig. 5a). The G_1_ offspring of the three heterozygous mutant (G_0_) adults were inbred for three generations to develop three lines, two of which were homozygous for a 20-bp insertion (*flot*^20+/20+^) or a 32-bp insertion (*flot*^32+/32+^) in the target site in G_4_. Western blot assays demonstrated that these insertions created frameshifts which resulted in the loss of the flotillin 2 protein (Fig. 5b). Due to the limited number of individuals in the *flot*^32+/32+^ line, we only used the *flot*^20+/20+^ line as the *flotillin 2*-knockout group in subsequent experiments. Compared with the wild-type planthoppers, the *flot*^20+/20+^ line did not show significant differences in survival rate throughout their whole life spans (Fig. 5c) and fecundity (Fig. 5d). Electrical penetration graph (EPG) analysis showed that feeding behaviors relative to viral transmission, i.e., pathway phases (waveforms N1, N2, N3), watery salivation (waveform N4-a), and passive ingestion (waveform N4-b), were not damaged in the *flotillin 2*-knockout planthoppers (Fig. 5e).

Compared to that in the wild-type planthoppers, fewer virions entered the cytoplasm of midgut epithelial cells after 12 h of oral acquisition from infected plants (Fig. 5f), and a much lower virus load was observed in epithelial cells at 15 dpi in the *flotillin 2*-knockout planthoppers (Fig. 5g). The amount of virus in the whole body was reduced by 56.9% according to the *NP* RNA level at 15 dpi (Fig. 5h). The disease incidence in the rice plants which were fed upon by the *flotillin 2*-knockout planthoppers was lower than that in the plants which were fed upon by the wild-type planthoppers. It was 11.7% in the *flotillin 2*-knockout group and 40% in the wild-type group at 22 dpi (Fig. 5i). Furthermore, the disease symptoms of plants were mitigated in the *flotillin 2*-knockout group (Fig. 5i). These results showed that flotillin 2 had a large influence on RSV transmission.

### The role of flotillin 2 in the infection of insect vectors by other rice viruses.

**FIG 6:**
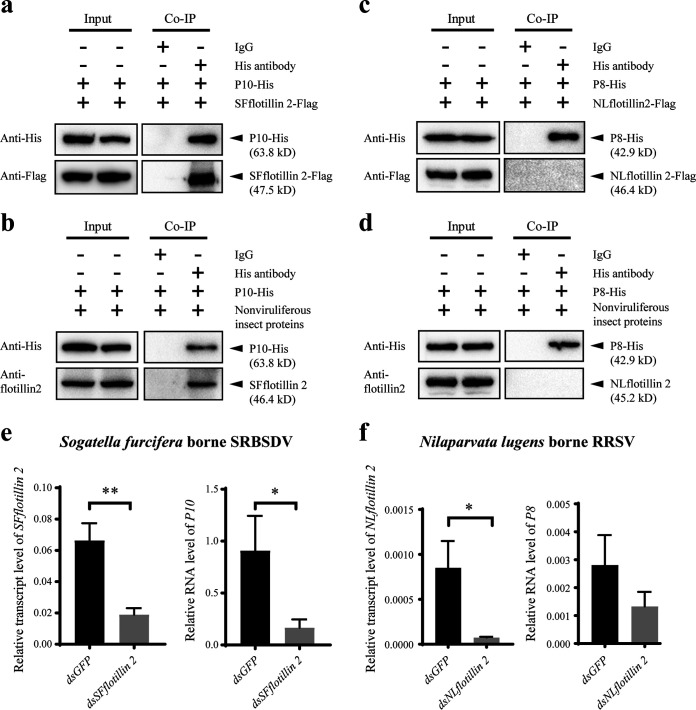
The role of flotillin 2 in the infection of insect vectors by other rice viruses. (a and b) The recombinantly expressed P10-His of SRBSDV was coprecipitated with SFflotillin 2-Flag (a) or SFflotillin 2 from the total protein of white-backed planthoppers (b) using the anti-His monoclonal antibody in Co-IP assays. Mouse IgG was used as negative control. The anti-His, anti-Flag, or anti-flotillin 2 antibodies were applied in Western blotting analyses. (c) and (d) The recombinantly expressed P8-His of RRSV was not coprecipitated with NLflotillin 2-Flag (c) or NLflotillin 2 from the total protein of brown planthoppers (d) in Co-IP assays. (e) Relative RNA levels of SRBSDV *P10* in the white-backed planthopper after 10 days of oral acquisition of SRBSDV with the injection of dsRNA for *SFflotillin 2* (dsSFflotillin 2) or dsRNA for *GFP* (dsGFP), as measured by qPCR. Relative transcription levels of *SFflotillin 2* were measured at the same time. Transcription level of ribosomal *L9* was quantified to normalize the cDNA template. (f) Relative RNA levels of RRSV *P8* in the brown planthopper after 10 days of oral acquisition of RRSV with the injection of dsNLflotillin 2 or dsGFP, as measured by qPCR. Relative transcription levels of *NLflotillin 2* were measured at the same time. Transcription level of 18S rRNA was quantified to normalize the cDNA template. *, *P* < 0.05; **, *P* < 0.01.

In addition to the interaction between small brown planthopper flotillin 2 and RSV NP, we predicted that brown planthopper flotillin 2 (NLflotillin 2) would interact with major capsid protein P8 of RRSV, and that white-backed planthopper flotillin 2 (SFflotillin 2) would interact with structural protein P10 of SRBSDV *in silico* (14). To clarify the roles of the homologous flotillin 2 proteins in the infection of the two planthopper species by RRSV or SRBSDV, we tested the interactions between SFflotillin 2 and P10 of SRBSDV and those between NLflotillin 2 and P8 of RRSV. The Co-IP results showed that P10 was able to bind to both recombinantly expressed SFflotillin 2-Flag and SFflotillin 2 from white-backed planthoppers (Fig. 6a and b), while P8 did not bind to recombinantly expressed NLflotillin 2-Flag or NLflotillin 2 from brown planthoppers (Fig. 6c and d). When the expression of *SFflotillin 2* was knocked down via the injection of ds*SFflotillin 2*-RNA, the level of SRBSDV *P10* RNA was dramatically decreased in white-backed planthoppers at 10 days after feeding on SRBSDV-infected rice plants (Fig. 6e). On the other hand, the knockdown of *NLflotillin 2* expression did not result in significant variation in RRSV levels in brown planthoppers in terms of *P8* RNA levels at 10 days after feeding on RRSV-infected rice plants (Fig. 6f). Therefore, flotillin 2 only mediates the infection of SRBSDV in the white-backed planthopper and not that of RRSV in the brown planthopper.

## DISCUSSION

Major barriers to systemic viral infection, such as the midgut infection barrier, are likely based on receptor recognition as a prerequisite for successful viral entry. This process requires specific interactions between virus components and the insect vector. We identified flotillin 2 as a key factor on the plasma membrane of midgut epithelial cells which facilitates the infection of a persistent-circulative plant virus in its vector insect, and the specific interaction between flotillin 2 and the viral NP has a large effect on vector competence. Although ubiquitous and highly conserved flotillin proteins have been shown to take part in various cellular processes, we report their function in transporting virions into cells for the first time. The mechanism of flotillin-mediated virus internalization could be similar to that of the protein uptake process, in which flotillin 2 works as a mediator to recruit virions to coated pits of the plasma membrane.

Flotillin 2 is one of the dominant factors for RSV entry into midgut epithelial cells. The knockout of *flotillin 2* resulted in a 57% reduction in RSV levels in planthoppers. This incomplete block implies the existence of other factors affecting virus entry. For example, sugar transporter 6 also plays a role in mediating the entrance of RSV into planthopper midgut cells (13). In a recent work, we discovered that importin α2 controls RSV entry into insect salivary gland cells (19). Clathrin-mediated endocytosis has been found to be involved in tomato yellow leaf curl virus (TYLCV) transport across the midgut barrier in its whitefly vector (20). However, clathrin does not seem to affect TYLCV entry into midgut cells; instead, it plays a role in viral release from midgut cells into the hemolymph (20). Such a multiple-route strategy can ensure the efficient transmission of plant viruses. In addition to the viral NP involved in cell entry, the glycoprotein NSvc2 of RSV has been reported to help the virus overcome midgut barriers (21). Whether NSvc2 takes part in flotillin-mediated cell entry deserves further exploration.

Flotillin 2 may function as a key factor in the infection of several rice viruses in their vector insects. We predicted potential conserved protein factors essential for the transmission of RSV, SRBSDV, RRSV, RBSDV, and RGSV by the small brown planthopper, brown planthopper, or white-backed planthopper (14). These five viruses exhibit nonenveloped virions (22–26). Flotillin 2 is one of the homologous planthopper proteins involved in all planthopper-virus interactions. Flotillin 2 of the small brown planthopper interacts with the nucleocapsid proteins of RSV and RBSDV (P10); flotillin 2 of the white-backed planthopper interacts with the structural protein of SRBSDV (P10); and flotillin 2 of the brown planthopper interacts with the nucleocapsid protein of RRSV (P8), but not with the nucleocapsid protein of RGSV (NCP). In this study, we demonstrated that flotillin 2 plays an important role in the infection of RSV and SRBSDV in their insect vectors. However, flotillin 2 does not seem to be involved in the transmission of RRSV and RGSV by the brown planthopper, showing divergent evolution in the roles of vector flotillin 2 in different plant viruses.

The two flotillins of planthoppers behave differently from their homologs in mammals. The flotillin domain mediates oligomerization to form homo- and heterotetramers in mammals. Oligomerization and the PHB domain are both required for efficient membrane association and incorporation into membrane microdomains (27). Furthermore, the stabilities of the two flotillin proteins are interdependent, so the absence of one leads to a reduction in the level of the other (16, 28). In contrast, although the two flotillins of planthoppers contain conserved PHB and flotillin domains, they do not bind to each other, preventing hetero-oligomerization. Flotillin 2 alone mediates RSV cell entry, using its flotillin domain to bind to viral NPs, and does not require the contribution of flotillin 1. Therefore, it is possible that the two flotillins function independently in the small brown planthopper.

Taking these results together, RSV exploits flotillin 2 to overcome the midgut barrier in infecting insect vectors. The deletion of *flotillin 2* can reduce vector competence for RSV and greatly suppress rice disease incidence. Therefore, *flotillin 2* would be a promising target gene for manipulation in insect vectors to control the transmission of rice stripe disease and perhaps that of other rice virus diseases in the future.

## MATERIALS AND METHODS

### Planthoppers and rice viruses.

The viruliferous and nonviruliferous small brown planthopper strains were reared separately in the laboratory on seedlings of rice, Oryza sativa Huangjinqing, at 25°C with 16 h of light daily, as described previously (29). The viruliferous strain harbored the Jiangsu RSV isolate, and the frequency of RSV positivity was maintained at more than 90% through purification selection performed every 3 months via dot-ELISA using a monoclonal anti-NP antibody (30). The nonviruliferous brown planthoppers and RRSV originated from Fuzhou, Fujian Province. The nonviruliferous white-backed planthoppers and SRBSDV originated from Guilin, Guangxi Province. The two species of planthoppers were maintained in chambers at 27°C with 16 h of light daily.

### RNA isolation and cDNA synthesis.

Total RNA was isolated from the whole bodies of planthoppers or from six tissues (brain, salivary gland, gut, fat body, ovary, and testicle) following the standard TRIzol reagent protocol (Invitrogen, Carlsbad, CA, USA). The concentration and quality of total RNA were determined using a NanoDrop 2000 spectrophotometer (Thermo Scientific, Waltham, MA, USA) and via gel electrophoresis. RNA was treated using DNase I (Qiagen, Valencia, CA, USA) to remove genomic DNA contamination before the DNA was used for cDNA synthesis. Two micrograms of total RNA from whole bodies, or 1 μg of total RNA from each tissue, was reverse transcribed to generate cDNA using the Superscript III First-Strand Synthesis System (Invitrogen, USA) and random primers (Promega, Madison, WI, USA) following the manufacturer’s instructions.

### Gene cloning and sequence analysis.

Based on genomic information for the three planthopper species (18, 31, 32), the ORFs of *flotillin 1* and *flotillin 2* from *L. striatellus*, and those of *flotillin 2* from *N. lugens* and *S. furcifera*, were amplified from the cDNA library for sequencing. RSV *NP*, RRSV *P8*, and SRBSDV *P10* were amplified from the cDNA library for sequencing. The primers will be provided upon request. The molecular weights of the encoded proteins were predicted in ExPASy (http://web.expasy.org/compute_pi/). The protein domain architectures of flotillin 1 and flotillin 2 were predicted with SMART (http://smart.embl-heidelberg.de/). The homologous protein sequences from other insect species, mice, and humans were aligned with flotillin 1 and flotillin 2 of the small brown planthopper, and an unrooted phylogenetic tree was constructed via the neighbor-joining method using the pairwise deletion and p-distance model in Mega 5.01 software. Bootstrap analysis with 1,000 replicates was performed to evaluate the internal support for the tree topology.

### Recombinant protein expression in *Escherichia coli*, purification, and antibody preparation.

The *flotillin 1* of *L. striatellus*, *flotillin 2* of *N. lugens* and *S. furcifera*, and *NP* ORFs were subcloned into the pET28a vector to generate recombinant plasmids with a Flag-tag. The ORF of *flotillin 2* of *L. striatellus* and its N terminus (1 to 86 aa), PHB domain (87 to 269 aa) and C terminus (270 to 424 aa), and the ORFs of *NP*, *P8*, and *P10*, were introduced into the pET28a vector to generate recombinant plasmids with a His tag. The ORF of *flotillin 2* of *L. striatellus* was inserted into the pMAL-c5X vector to generate the recombinant plasmid with a maltose-binding protein (MBP)-tag. *NP* ORF was subcloned into the pGEX-3× vector to generate the recombinant plasmid with a GST-tag. The primers will be provided upon request. The recombinant plasmids were transformed into Escherichia coli strains BL21 or Transetta (DE3) for protein expression. After 2 h of induction with 1 mM isopropyl β-d-thiogalactoside (IPTG) at 37°C, the cells were pelleted by centrifugation and sonicated for 30 min in ice water. The supernatant was retained for coimmunoprecipitation assays or protein purification. The flotillin 2-His of *L. striatellus* was purified from the supernatant using Ni Sepharose (GE Healthcare, Buckinghamshire, UK) with 100 mM imidazole, following the manufacturer’s instructions, and dissolved in 10 mM Tris-HCl (pH 8.0) after filtration with a 10-kDa cutoff Amicon Ultra Centrifugal Filter (Millipore, Burlington, MA, USA). The purified flotillin 2-His served as the antigen for producing an anti-flotillin 2 rabbit polyclonal antibody at the Beijing Protein Institute Co., Ltd. (Beijing, China). The specificity of the anti-flotillin 2 antibody was tested for the recognition of *in vitro*-expressed flotillin 2-His and flotillin 1-Flag proteins and the flotillin proteins within the total planthopper protein through Western blot analysis. The flotillin 2-MBP, NP-GST and GST were purified using Amylose Resin (NEB, Ipswich, MA, USA) or glutathione Sepharose 4B beads (GE Healthcare) following the manufacturers’ instructions.

### Co-IP assay.

Ten micrograms of an NP monoclonal antibody were first incubated with 50 μL of Dynabeads Protein G (Novex, Thermo Fisher Scientific, Waltham, MA, USA) for 30 min at room temperature, after which 400 μL of the total protein from viruliferous *L. striatellus* in 10 mM phosphate buffer (pH 7.4) was added, and incubation was performed for 2 h at 4°C. Approximately 10% of the total protein was reserved as input. Mouse IgG (Merck Millipore, Billerica, MA, USA) was used as a negative control. In another group, 10 μg of a mouse anti-Flag monoclonal antibody was first incubated with 50 μL of Dynabeads Protein G for 30 min, after which 400 μL of recombinantly expressed flotillin 1-Flag, P10-His, or P8-His was added, and incubation was performed for 2 h at 4°C. After washing three times with washing buffer (Novex), 400 μL of the total protein from viruliferous *L. striatellus*, or from nonviruliferous *S. furcifera* or *N. lugens*, was added, followed by another 2 h of incubation. The total protein from E. coli expressing empty pET28a or mouse IgG was applied in the control groups. After washing three times with washing buffer, the antibody-protein complex was disassociated from the beads with elution buffer (Novex) for Western blot analysis using anti-NP, anti-flotillin 2, anti-His, or anti-Flag antibodies.

Five micrograms of a mouse anti-His monoclonal antibody or a mouse anti-Flag monoclonal antibody were incubated with 50 μL of Dynabeads Protein G (Novex) for 15 min at room temperature. Then, 400 μL of a 1:1 mixture of recombinantly expressed NP-Flag and each flotillin 2 fragment (N-terminal-His, PHB-His or C-terminal-His); a 1:1 mixture of flotillin 1-Flag of *L. striatellus* and NP-His; a 1:1 mixture of flotillin 1-Flag and flotillin 2-His of *L. striatellus*; a 1:1 mixture of flotillin 2-Flag of *S. furcifera* and P10-His; or a 1:1 mixture of flotillin 2-Flag of *N. lugens* and P8-His was added, and incubation was performed for 2 h at 4°C. The total protein from E. coli expressing empty pET28a or mouse IgG was applied in the control groups. After washing three times with washing buffer (Novex), the antibody-protein complex was disassociated from the beads with elution buffer (Novex) for Western blot analysis with anti-Flag or anti-His monoclonal antibodies (CWBiotech, Beijing, China).

### Immunohistochemistry.

Midguts were dissected from nonviruliferous or viruliferous fourth- or fifth-instar nymphs of *L. striatellus* in cold 1× PBS buffer (0.01 M phosphate-buffered saline [pH 7.4]) on a glass plate and fixed in 4% paraformaldehyde for 2 h at room temperature. The samples were permeabilized with 1× PBST buffer (0.01 M phosphate-buffered saline containing 1% Triton X-100 [pH 7.4]) for 2 h and then blocked with 5% bovine serum albumin (BSA) for 30 min at room temperature. The samples were incubated with the anti-flotillin 2 polyclonal antibody overnight at 4°C, either alone or followed by incubation with the anti-NP monoclonal antibody for 2 h. After washing with 1× PBST buffer, the secondary antibodies Alexa Fluor 488 (green) AffiniPure goat anti-mouse IgG and Alexa Fluor 594 (red) AffiniPure goat anti-rabbit IgG (Invitrogen) were added. Phalloidin was used to label F-actin (blue) in accordance with the manufacturer’s instructions (Abcam, Cambridge, UK). The negative control did not include the primary antibodies. The results were viewed under a Zeiss LSM 710 confocal microscope (Carl Zeiss AG, Oberkochen, Germany).

The full-length flotillin 2 of *L. striatellus* and the three fragments thereof (N-terminal, PHB, and C-terminal) were introduced into the pAC 5.1B vector with a His-tag. The primers wcanill be provided upon request. The recombinant plasmids (200 ng/well) were transfected into 500 μL of *Drosophila* S2 cells in a 24-well plate using Lipofectamine 3000 (Invitrogen). After 48 h, the cells were fixed with 4% paraformaldehyde for 30 min at room temperature. After washing three times with PBS buffer and twice with distilled water for 5 min each time, the cells were blocked with 1% BSA for 30 min and then sequentially incubated with the anti-His monoclonal antibodies (CWBiotech) at 4°C overnight and the secondary antibody Alexa Fluor 488 AffiniPure goat anti-mouse IgG (Invitrogen) for 1 h at room temperature. After washing three times with 1× PBST buffer, the nuclei were stained with Hoechst, and F-actin was labeled with phalloidin (Abcam). The negative control did not include the primary antibody. The results were viewed under a Zeiss LSM 710 confocal microscope (Carl Zeiss AG).

### Colloidal gold immunoelectron microscopy.

Midguts were dissected from viruliferous fourth-instar nymphs of *L. striatellus* and fixed with 2% paraformaldehyde and 2% glutaraldehyde in 0.1 M PBS buffer (pH 7.2) for 2 h at room temperature. After dehydration in 30, 50, 70, 90, 95 and 100% alcohol, the samples were embedded with LR White Resin (Fluka Biochemika, Steinheim, Switzerland) and polymerized with a Leica EM AFS2 system (Leica Microsystems, Solms, Germany). Then, 60-nm ultrathin sections of the embedded samples were cut using a Leica EM FC7 ultramicrotome (Leica Microsystems) and attached to 50-mesh copper grids (Electron Microscopy Sciences, Fort Washington, PA, USA) before being blocked for 30 min in 0.1 M PBS (pH 7.4) containing 5% bovine serum albumin. The anti-NP mouse monoclonal antibody (1:100) and the anti-flotillin 2 rabbit polyclonal antibody (1:100) were sequentially added, followed by 1.5 h of incubation each time. Then, 10-nm gold-conjugated goat-anti-mouse IgG (1:200) and 5-nm gold-conjugated goat-anti-rabbit IgG (1:200) were added, followed by 1 h of incubation at room temperature. After being washed four times with double-distilled water, the sections were stained with 2% uranyl acetate for 10 min in the dark and viewed with a transmission electron microscope (Tecnai Spirit TEM, FEI, Lausanne, Switzerland) at 100 kV. Samples that were not subjected to primary antibody treatment were used as negative controls.

### MST and BLI assays.

The affinity between flotillin 2-MBP and NP-GST was measured with MST assay in a Monolith NT.115 instrument (NanoTemper Technologies, Munich, Germany). Flotillin 2-MBP protein was labeled with the blue fluorescent dye NHS-495. The concentration of flotillin 2-MBP was held constantly at 10 μM, whereas the concentrations of NP-GST were gradient-diluted from 17.5 μM to 0.0417 nM. After a brief incubation, the samples were loaded into MST-standard glass capillaries. The measurements were performed at 25°C. The purified GST protein was used instead of NP-GST in the control group. Data were analyzed to obtain the dissociation constant (*K*_D_) using software Monolith Affinity Analysis v2.2.4.

The binding affinity of flotillin 2-MBP to NP-GST was also analyzed with a BLI assay using the Octet RED96e system (FortéBio, Fremont, CA, USA). The experiment was performed at 25°C in a buffer containing 20 mM NaH_2_PO_4_, 150 mM NaCl, and 0.05% Tween 20. Streptavidin biosensors were pre-equilibrated in buffer for at least 10 min before use. Biotinylated flotillin 2-MBP (5 μM) was loaded onto streptavidin biosensors for 180 s to immobilize. The association curve was determined by flowing through various concentrations of NP-GST proteins, from 0 to 4,000 nM, in 1× PBS buffer. After association, the tips were dipped back into 1× PBS to obtain the dissociation curve. The binding kinetics (*K*_D_), association rate (*K*_on_), and dissociation rate (*K*_off_) were calculated using Octet data analysis software.

### Quantification of two *flotillin* gene expressions in viruliferous and nonviruliferous *L. striatellus*.

The transcription levels of *flotillin 1* and *flotillin 2* were quantified in two groups of *L. striatellus* using qPCR. The primers will be provided upon request. One group was the fourth-instar nymphs of the viruliferous and nonviruliferous strains. The other group was the fourth-instar nymphs of nonviruliferous planthoppers fed on RSV-infected rice seedlings or healthy rice seedlings for 8 days. Five insects were included in each replicate, and six replicates were prepared for each group. The transcription levels of *flotillin 2* were quantified using qPCR in the brain, salivary gland, gut, fat body, ovary, and testicle tissues collected from 20 to 50 nonviruliferous adult planthoppers. Six replicates were prepared for each tissue.

### Injection of dsRNA and feeding planthoppers with RSV-infected rice seedlings.

dsRNAs for *flotillin 1* (158 bp), *flotillin 2* (129 bp), and *GFP* (420 bp) were synthesized using the T7 RiboMAX Express RNAi System (Promega) following the manufacturer’s protocol. The primers of the dsRNAs for these genes will be provided upon request. Nonviruliferous third-instar nymphs of *L. striatellus* fed on RSV-infected rice seedlings for 4 days, and then 23 nL of dsRNAs of *flotillin 1* or *flotillin 2* at 6 μg/μL was injected into planthoppers using a Nanoliter 2000 microinjector (World Precision Instruments, Sarasota, FL, USA). The insects were raised on healthy rice for 6 days prior to collection for the quantification of viral *NP* RNA levels and transcription levels of *flotillin 1* and *flotillin 2* using qPCR. Five insects were included in each replicate, and six replicates were prepared. The level of NP was measured using the anti-NP monoclonal antibody, and an anti-human β-tubulin monoclonal antibody (EASYBIO, Beijing, China) was used to measure tubulin as an internal control in Western blot assays. Three replicates were performed for each group. The midguts of insects were dissected for immunohistochemistry assays using the anti-flotillin 2 polyclonal antibody and the anti-NP monoclonal antibody.

In another experiment, nonviruliferous fourth-instar nymphs of *L. striatellus* were injected with 23 nL of dsRNAs of *flotillin 2* or *GFP* at 6 μg/μL. After 3 days, the insects fed on RSV-infected rice seedlings for 12 h were collected for midgut dissection and the following immunohistochemistry assays using the anti-flotillin 2 polyclonal antibody and the anti-NP monoclonal antibody.

### Knockout of flotillin 2 in *L. striatellus* using CRISPR-Cas9.

We applied CasOT (33) to search sgRNAs and potential off-target sites in the small brown planthopper genome using the *flotillin 2* sequence. The search criteria for sgRNAs were a 20-nucleotide length and an NGG motif at the 3′-end (protospacer-adjacent motif [PAM]) of the target site. The maximum numbers of mismatches allowed in the seed and nonseed regions were 2 and 3, respectively. In off-target mode, the two parameters included the types of sites allowed (NGG at PAM and G at the 5′-end) and the allowed length range of the protospacers of the candidate sites (19 to 20 nt by default). The sgRNA with the lowest off-target possibility was predicted to target the first 20 bp of the *flotillin 2* ORF. The sgRNA was synthesized using the GeneArt Precision gRNA Synthesis Kit (Invitrogen) following the manufacturer’s instructions. The primers will be provided upon request.

Eggs were dissected from rice sheathes within 1 h of oviposition and lined up on 2% agar containing 0.005‰ methylene blue in a 9 cm-diameter petri dish. A mixture of 2.3 nL of 40 ng/μL sgRNA and 133 ng/μL GeneArt Platinum Cas9 protein (Invitrogen) was injected into the posterior portion of individual eggs using a Nanoliter 2000 microinjector (World Precision Instruments) within 3 h of oviposition. After injection, the petri dishes were placed in a chamber at 23°C with 16 h of light daily. The hatched first-instar nymphs were transferred to fresh rice seedlings. At 48 h after injection, genomic DNA was extracted from individual eggs using the cetyltrimethylammonium bromide (CTAB) method as described previously (34). The PCR products spanning sgRNA target sites were amplified and subcloned into the pLB vector (Tiangen Company, Beijing, China) for Sanger sequencing to examine mutations. The primers will be provided upon request. G_0_ adults were mated with wild-type planthoppers. After obtaining G_1_ eggs, genomic DNA was extracted from individual G_0_ adults using the CTAB method. The PCR products spanning the sgRNA target sites were amplified for Sanger sequencing. The G_1_ offspring of the mutant G_0_ adults were inbred for three generations, and the genotypes of the parents, spanning the sgRNA target sites in each generation, were checked as described above. Finally, *flotillin 2* homozygous mutant lines were successfully established. The flotillin 2 levels in the mutant lines and in the wild-type planthoppers were assayed by Western blotting using an anti-flotillin 2 polyclonal antibody. An anti-human β-tubulin monoclonal antibody (EASYBIO, Beijing, China) was used to measure tubulin as an internal control.

### Survival curves and fecundity of wild-type or flotillin 2-knockout planthoppers.

Sixty first-instar nymphs of wild-type or flotillin 2-knockout planthoppers were collected within 6 h after hatching and individually raised on five rice seedings. Survival of each insect from birth to death was recorded daily. Survival curves were assessed by the Kaplan-Meier method and difference was evaluated according to the Mantel-Cox log-rank statistic test. For fecundity measurement, single mated females were raised on five rice seedings and eggs were collected within 10 days. The average numbers of eggs from 18 wild-type and 20 flotillin 2-knockout females were compared using Student's *t* test.

### EPG analysis of small brown planthopper feeding behavior.

The EPG waveforms of feeding behavior of wild-type and flotillin 2-knockout planthoppers were recorded continuously for 4 h using a Giga-8 EPG amplifier (Wageningen University, Wageningen, Netherlands) according to a previously described procedure (35). The waveforms of non-probing (np), pathway phases (N1+N2+N3), watery salivation in phloem (N4-a), passive ingestion in phloem (N4-b), and drinking from xylem (N5) from 22 individuals were analyzed with the software Stylet+a and their time ratios were reported as mean ± standard error of the mean (SE). Differences between the two groups were statistically analyzed using the Mann-Whitney U test in SigmaPlot 12.0.

### Feeding wild-type or flotillin 2-knockout planthoppers with RSV-infected rice seedlings.

Nonviruliferous third-instar nymphs of the wild-type or the flotillin 2-knockout strain of *L. striatellus* fed on RSV-infected rice seedlings for 12 h or 15 days. The levels of *NP* RNA in single insects were measured using qPCR, and 14 individuals were checked in each group. The midguts of the insects were dissected for immunohistochemistry assays using the anti-flotillin 2 polyclonal antibody and the anti-NP monoclonal antibody.

### Disease incidence rates of rice plants.

Nonviruliferous third-instar nymphs of the wild-type or flotillin 2-knockout planthoppers fed on RSV-infected rice seedlings for 8 days and then transferred to new healthy rice seedlings to feed for 2 days, after which the insects were removed from the plants. The plants were cultured in a greenhouse at 30°C with 16 h of light per day to observe disease symptoms. Ten plants per replicate and five or six replicates were used to calculate disease incidence rates on each day.

### Injection of dsRNAs and feeding white-backed planthoppers and brown planthoppers with SRBSDV and RRSV.

dsRNAs for *SFflotillin 2* (172 bp) and *NLflotillin 2* (209 bp) were synthesized using the T7 RiboMAX Express RNAi System (Promega). The primers used for dsRNA synthesis will be provided upon request. Aliquots of 23 nL of 6 μg/μL dsRNAs were injected into nonviruliferous fourth-instar nymphs of white-backed or brown planthoppers. After injection, the planthoppers fed on SRBSDV- or RRSV-infected rice for 4 days, then reared on new healthy rice seedlings for 6 days. Then, the insects were collected for the quantification of RNA levels of SRBSDV *P10* or RRSV *P8*, and transcription levels of *SFflotillin 2* or *NLflotillin 2* using qPCR. The primers will be provided upon request. The control group was injected with ds*GFP*-RNA. Five insects were included in each replicate, and six to eight replicates were prepared for each group.

### qPCR.

qPCR was used to measure the relative RNA levels of viral and planthopper genes on a Light Cycler 480 II instrument (Roche, Basel, Switzerland). The transcription levels of *translation elongation factor 2* (*EF2*) for the small brown planthopper, 18S rRNA for the brown planthopper, and *ribosomal L9* for the white-backed planthopper were quantified to normalize the cDNA template. The primers will be provided upon request. qPCR was performed in a 20-μL volume comprising 1 μL of template cDNA, 10 μL of 2× SYBR Green PCR Master Mix (Fermentas, Waltham, MA, USA), and 0.25 μL of each primer (10 μM). The thermal cycling conditions were 95°C for 5 min, followed by 40 cycles of 94°C for 20 s, 60°C for 30 s, and 72°C for 10 s. The transcription levels of each gene relative to that of *EF2*, 18S rRNA, or *ribosomal L9* was reported as the mean ± SE. Differences were statistically evaluated using Student's *t* test to compare two means and one-way analysis of variance followed by Tukey’s test for multiple comparisons in SPSS v17.0.

## References

[1] Hohn T. 2007. Plant virus transmission from the insect point of view. Proc Natl Acad Sci USA 104:17905–17906. 10.1073/pnas.0709178104. 17989216 PMC2084268

[2] Bragard C, Caciagli P, Lemaire O, Lopez-Moya JJ, MacFarlane S, Peters D, Susi P, Torrance L. 2013. Status and prospects of plant virus control through interference with vector transmission. Annu Rev Phytopathol 51:177–201. 10.1146/annurev-phyto-082712-102346. 23663003

[3] Hogenhout SA, Ammar el D, Whitfield AE, Redinbaugh MG. 2008. Insect vector interactions with persistently transmitted viruses. Annu Rev Phytopathol 46:327–359. 10.1146/annurev.phyto.022508.092135. 18680428

[4] Toriyama S. 1986. Rice stripe virus: prototype of a new group of viruses that replicate in plants and insects. Microbiol Sci 3:347–351. 2856619

[5] Wei T-Y, Yang J-G, Liao F-L, Gao F-L, Lu L-M, Zhang X-T, Li F, Wu Z-J, Lin Q-Y, Xie L-H, Lin H-X. 2009. Genetic diversity and population structure of rice stripe virus in China. J Gen Virol 90:1025–1034. 10.1099/vir.0.006858-0. 19264655

[6] Zhou Y, Li S, Cheng Z, Zhou T, Fan Y. 2012. Research advances in rice stripe disease in China. Jiangsu J Agric Sci 28:1007–1015.

[7] Hamamatsu C, Toriyama S, Toyoda T, Ishihama A. 1993. Ambisense coding strategy of the rice stripe virus genome: *in vitro* translation studies. J Gen Virol 74:1125–1131. 10.1099/0022-1317-74-6-1125. 8509762

[8] Zhao W, Xu Z, Zhang X, Yang M, Kang L, Liu R, Cui F. 2018. Genomic variations in the 3'-termini of rice stripe virus in the rotation between vector insect and host plant. New Phytol 219:1085–1096. 10.1111/nph.15246. 29882354 PMC6055815

[9] Wang W, Zhao W, Li J, Luo L, Kang L, Cui F. 2017. The c-Jun N-terminal kinase pathway of a vector insect is activated by virus capsid protein and promotes viral replication. Elife 6:e26591. 10.7554/eLife.26591. 28716183 PMC5515582

[10] Chen X, Yu J, Wang W, Lu H, Kang L, Cui F. 2020. A plant virus ensures viral stability in the hemolymph of vector insects through suppressing prophenoloxidase activation. mBio 11:e01453-20. 10.1128/mBio.01453-20. 32817105 PMC7439478

[11] Zhao W, Yu J, Jiang F, Wang W, Kang L, Cui F. 2021. Coordination between terminal variation of the viral genome and insect microRNAs regulates rice stripe virus replication in insect vectors. PLoS Pathog 17:e1009424. 10.1371/journal.ppat.1009424. 33690727 PMC7984632

[12] Zhao W, Zhu JJ, Lu H, Zhu JM, Jiang F, Wang W, Luo L, Kang L, Cui F. 2021. The nucleocapsid protein of rice stripe virus in cell nuclei of vector insect regulates viral replication. Protein Cell 6:1–19. 10.1007/s13238-021-00822-1. PMC7936609 33675514

[13] Qin FL, Liu WW, Wu N, Zhang L, Zhang ZK, Zhou XP, Wang XF. 2018. Invasion of midgut epithelial cells by a persistently transmitted virus is mediated by sugar transporter 6 in its insect vector. PLoS Pathog 14:e1007201. 10.1371/journal.ppat.1007201. 30052679 PMC6082570

[14] Zhu J, Eid FE, Tong L, Zhao W, Wang W, Heath LS, Kang L, Cui F. 2021. Characterization of protein-protein interactions between rice viruses and vector insects. Insect Sci 28:976–986. 10.1111/1744-7917.12840. 32537916

[15] Morrow IC, Parton RG. 2005. Flotillins and the PHB domain protein family: rafts, worms and anaesthetics. Traffic 6:725–740. 10.1111/j.1600-0854.2005.00318.x. 16101677

[16] Frick M, Bright NA, Riento K, Bray A, Merrified C, Nichols BJ. 2007. Coassembly of flotillins induces formation of membrane microdomains, membrane curvature, and vesicle budding. Curr Biol 17:1151–1156. 10.1016/j.cub.2007.05.078. 17600709

[17] Doherty GJ, McMahon HT. 2009. Mechanisms of endocytosis. Annu Rev Biochem 78:857–902. 10.1146/annurev.biochem.78.081307.110540. 19317650

[18] Zhu J, Jiang F, Wang X, Yang P, Bao Y, Zhao W, Wang W, Lu H, Wang Q, Cui N, Li J, Chen X, Luo L, Yu J, Kang L, Cui F. 2017. Genome sequence of the small brown planthopper, *Laodelphax striatellus*. Gigascience 6:1–12. 10.1093/gigascience/gix109. PMC5740986 29136191

[19] Ma Y, Lu H, Wang W, Zhu J, Wan Z, Cui F. 2021. Membrane association of importin α facilitates viral entry into salivary gland cells of vector insects. Proc Natl Acad Sci USA 118:e2103393118. 10.1073/pnas.2103393118. 34290144 PMC8325321

[20] Pan LL, Chen QF, Zhao JJ, Guo T, Wang XW, Hariton-Shalev A, Czosnek H, Liu SS. 2017. Clathrin-mediated endocytosis is involved in Tomato yellow leaf curl virus transport across the midgut barrier of its whitefly vector. Virology 502:152–159. 10.1016/j.virol.2016.12.029. 28056414

[21] Lu G, Li S, Zhou C, Qian X, Xiang Q, Yang T, Wu J, Zhou X, Zhou Y, Ding XS, Tao X. 2019. Tenuivirus utilizes its glycoprotein as a helper component to overcome insect midgut barriers for its circulative and propagative transmission. PLoS Pathog 15:e1007655. 10.1371/journal.ppat.1007655. 30921434 PMC6456217

[22] Shikata E, Kitagawa Y. 1977. Rice black-streaked dwarf virus: its properties, morphology and intracellular localization. Virology 77:826–842. 10.1016/0042-6822(77)90502-5. 855190

[23] Hibino H, Usugi T, Omura T, Tsuchizaki T, Shohara K, Iwasaki M. 1985. Rice grassy stunt virus: a planthopper-borne circular filament. Phytopathology 75:894–899. 10.1094/Phyto-75-894.

[24] Hagiwara K, Minobe Y, Nozu Y, Hibino H, Kimura I, Omura T. 1986. Component proteins and structure of rice ragged stunt virus. J Gen Virol 67:1711–1715. 10.1099/0022-1317-67-8-1711.

[25] Zhou GH, Wen JJ, Cai DJ, Li P, Xu DL, Zhang SG. 2008. Southern rice black-streaked dwarf virus: a new proposed Fijivirus species in the family Reoviridae. Chin Sci Bull 53:3677–3685. 10.1007/s11434-008-0467-2.

[26] Whitfield AE, Falk BW, Rotenberg D. 2015. Insect vector-mediated transmission of plant viruses. Virology 479–480:278–289. 10.1016/j.virol.2015.03.026. 25824478

[27] Solis GP, Hoegg M, Munderloh C, Schrock Y, Malaga-Trillo E, Rivera-Milla E, Stuermer CAO. 2007. Reggie/flotillin proteins are organized into stable tetramers in membrane microdomains. Biochem J 403:313–322. 10.1042/BJ20061686. 17206938 PMC1874235

[28] Babuke T, Ruonala M, Meister M, Amaddii M, Genzler C, Esposito A, Tikkanen R. 2009. Hetero-oligomerization of reggie-1/flotillin-2 and reggie-2/flotillin-1 is required for their endocytosis. Cell Signal 21:1287–1297. 10.1016/j.cellsig.2009.03.012. 19318123

[29] Zhao W, Lu L, Yang P, Cui N, Kang L, Cui F. 2016a. Organ-specific transcriptome response of the small brown planthopper toward rice stripe virus. Insect Biochem Mol Biol 70:60–72. 10.1016/j.ibmb.2015.11.009. 26678499

[30] Zhao W, Yang P, Kang L, Cui F. 2016b. Different pathogenicities of Rice stripe virus from the insect vector and from viruliferous plants. New Phytol 210:196–207. 10.1111/nph.13747. 26585422 PMC5063192

[31] Xue J, Zhou X, Zhang C-X, Yu L-L, Fan H-W, Wang Z, Xu H-J, Xi Y, Zhu Z-R, Zhou W-W, Pan P-L, Li B-L, Colbourne JK, Noda H, Suetsugu Y, Kobayashi T, Zheng Y, Liu S, Zhang R, Liu Y, Luo Y-D, Fang D-M, Chen Y, Zhan D-L, Lv X-D, Cai Y, Wang Z-B, Huang H-J, Cheng R-L, Zhang X-C, Lou Y-H, Yu B, Zhuo J-C, Ye Y-X, Zhang W-Q, Shen Z-C, Yang H-M, Wang J, Wang J, Bao Y-Y, Cheng J-A. 2014. Genomes of the rice pest brown planthopper and its endosymbionts reveal complex complementary contributions for host adaptation. Genome Biol 15:521. 10.1186/s13059-014-0521-0. 25609551 PMC4269174

[32] Wang L, Tang N, Gao X, Chang Z, Zhang L, Zhou G, Guo D, Zeng Z, Li W, Akinyemi IA, Yang H, Wu Q. 2017b. Genome sequence of a rice pest, the white-backed planthopper (*Sogatella furcifera*). Gigascience 6:1–9. 10.1093/gigascience/gix068. PMC5437944 28369349

[33] Xiao A, Cheng Z, Kong L, Zhu Z, Lin S, Gao G, Zhang B. 2014. CasOT: a genome-wide Cas9/gRNA off-target searching tool. Bioinformatics 30:1180–1182. 10.1093/bioinformatics/btt764. 24389662

[34] Roger S, Bendich A. 1989. Extraction of DNA from plant tissues, p 73–83. In Gelvin SB, Schilperoort RA, Verma DPS (ed). Plant Molecular Biology Manual, Springer, Dordrecht, the Netherlands.

[35] Sun Z, Yan F, Wang MQ. 2017. Transgenic expression of *Bt* in rice does not affect feeding behavior and population density of the brown planthopper, *Nilaparvata lugens* Stål (Hemiptera: Delphacidae). Entomologia Generalis 37:35–45. 10.1127/entomologia/2017/0068.

